# The United States Army Aeromedical Research Laboratory Multi-Attribute Task Battery

**DOI:** 10.3389/fnrgo.2024.1435588

**Published:** 2024-12-18

**Authors:** Jonathan Vogl, Charles D. McCurry, Sharon Bommer, J. Andrew Atchley

**Affiliations:** ^1^United States Army Aeromedical Research Laboratory, Warfighter Performance Group, Fort Novosel, AL, United States; ^2^Department of Energy, Oak Ridge Institute for Science and Education, Oak Ridge, TN, United States; ^3^Goldbelt Frontier, LLC, Alexandria, VA, United States

**Keywords:** MATB, workload, Multi-Attribute Task Battery, automation, performance modeling

## Abstract

The U.S. Army Aeromedical Research Laboratory (USAARL) Multi-Attribute Task Battery (MATB) represents a significant advancement in research platforms for human performance assessment and automation studies. The USAARL MATB builds upon the legacy of the traditional MATB, which has been refined over 30 years of use to include four primary aviation-like tasks. However, the USAARL MATB takes this foundation and enhances it to meet the demands of contemporary research, particularly in the areas of performance modeling, cognitive workload assessment, adaptive automation, and trust in automation. The USAARL MATB retains the four classic subtask types from its predecessors while introducing innovations such as subtask variations, dynamic demand transitions, and performance-driven adaptive automation handoffs. This paper introduces the USAARL MATB to the research community, highlighting its development history, key features, and potential applications.

## 1 Introduction

Aviation research is highly involved in examining cognitive performance and human-machine interaction. Few research institutions have abundant access to expert-trained pilots who can provide the necessary skillset to interact with highly complex aviation components. Beyond that, few aviation simulation platforms are freely available while also providing research-quality data output. Combined, these factors limit researchers in their ability to assess cognitive performance on tasks that pilots face on a day-to-day basis.

The Multi-Attribute Task Battery (MATB) program has filled this gap by providing an aviation-like multitasking simulation platform to researchers. The MATB is considered aviation-like as it utilizes four simplified subtasks that are commonly seen in the aviation environment. One benefit of using the MATB is that non-pilots can easily understand and perform the subtasks, lessening the need to recruit experienced aviators. The MATB has been utilized as a research tool over the past 30 years. Although it has evolved throughout the years, the four subtasks and workload assessment used when the MATB was debuted are largely unchanged in its most current iteration.

### 1.1 USAARL MATB subtasks

**Figure 1:**
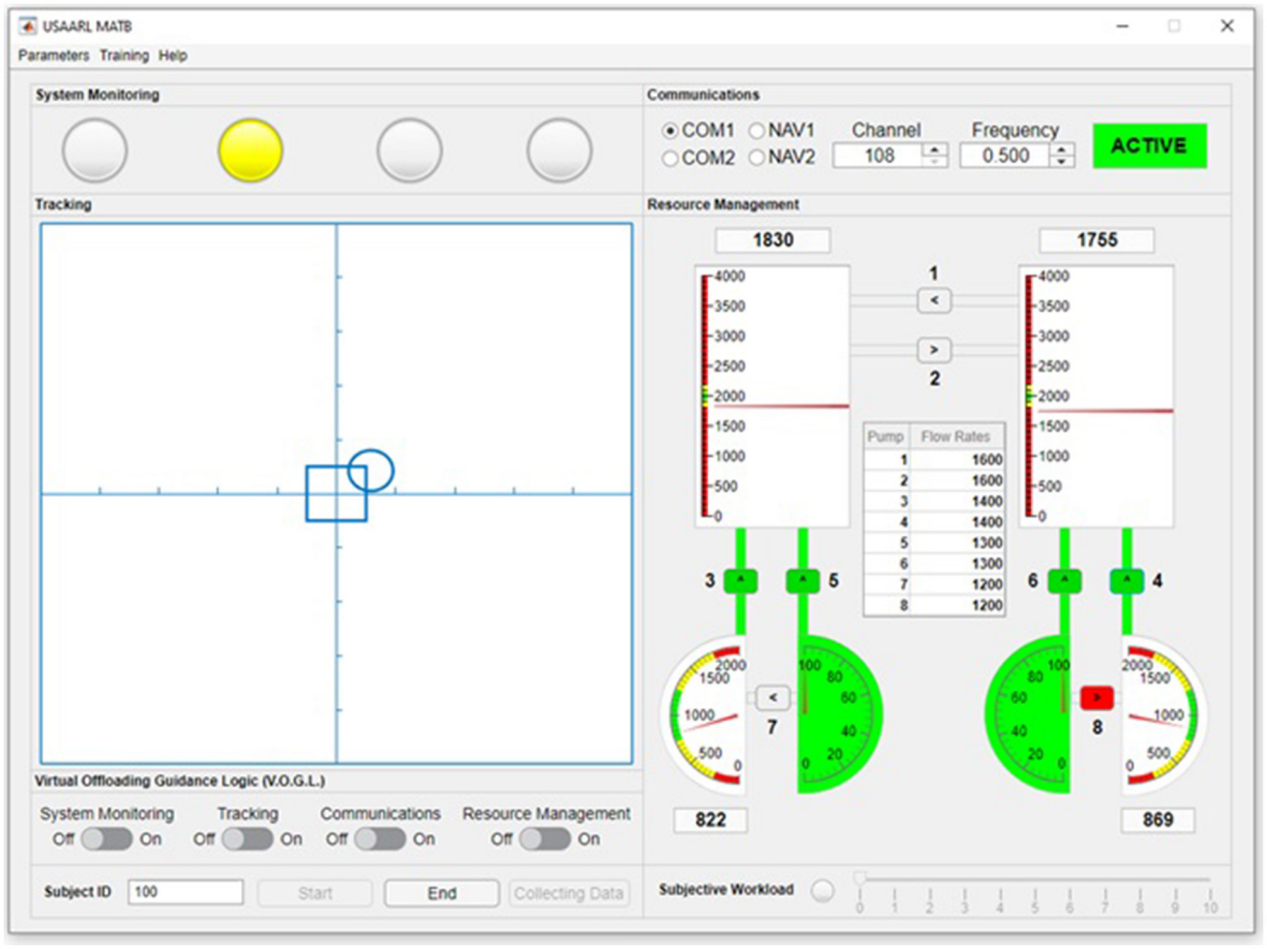
The USAARL MATB GUI. Additional display options exist, such as separating each task into their own windows and altering the size of interactive components to be more conducive to touch screen interactivity.

The MATB classically consists of four primary subtasks that are performed simultaneously during the simulation (Comstock and Arnegard, 1992). These four subtasks are referred to as the systems monitoring, communications, tracking, and resource management tasks. The MATB also typically includes a measure of cognitive workload. In this section, we provide an overview of the operator's goal for each task. The four subtasks as they are shown in the USAARL MATB can be seen in Figure 1. The components associated with each subtask are discussed in detail.

#### 1.1.1 System monitoring

The system monitoring task is a discrete visual vigilance task. Operators are tasked with monitoring the status of four round lights within the system monitoring panel. These lights correspond to buttons on a joystick relative to their position. Button 1 corresponds with the left most light, button 2 with the second from the leftmost light, etc. These lights have two states, either “On,” as indicated by it changing to a color (specified by the experimenter in the parameter file) from the background panel, or “Off,” as indicated by it matching the color of the background panel. When a light turns on, the operator presses the corresponding button on the joystick to turn the light off. If the operator does not press the corresponding button, the light will remain on for a pre-defined period. The timeout period is defined by the researcher during parameter generation. Both the reaction time and accuracy of the button press are utilized to score operator performance.

#### 1.1.2 Communications

The communications task is a discrete auditory vigilance task. For the communications task, the subject is tasked with following instructions from an audio stimulus dictating the radio, channel, and frequency options they must select. An example of a typical audio stimulus for the communications task is “NASA 504, NASA 504, turn your NAV1 radio to 126.475.” The audio stimuli used are the same files used in the NASA MATB-II (Santiago-Espada et al., 2011). There are three primary interactive components to the communications task that can be selected using the mouse, radio selection, channel selection, and frequency selection.

The first instruction of the audio stimulus asks the operator to change their radio to a specific radio name. Selecting the radio button to the left of the radio name will select that radio. The second instruction of the audio stimulus asks the operator to change the channel to a specific number. The channel consists of the first three numbers spoken by the speaker. These are numbers spoken before the word “point,” i.e., before the decimal. The operator can change the channel by pressing the channel spinner up or down to obtain the correct number or by typing it into the channel field. The third instruction of the audio stimulus asks the operator to change the frequency to a specific number. The second set of three numbers following the word “point,” i.e., the decimal, indicate the frequency value. The operator can select the frequency value by pressing the buttons on the spinner until the correct value is obtained or type them in manually.

When an auditory stimulus plays, the active light will change color to indicate that an auditory stimulus is playing. The active light serves as an indication for the operator to enter a response to the communications task. The active light will remain on for the duration of the audio stimulus and a pre-defined timeout period following the stimulus. While the active button is lit, the operator may input their response. Once a correct response is entered or once the timeout value is reached, the active light will turn off.

#### 1.1.3 Tracking

The tracking task is a continuous compensatory tracking task. A tracking grid with a centralized square and randomly moving circle are presented to the operator. The operator can influence the position of the circle by moving the joystick. Throughout the simulation, the circle will randomly drift across the tracking grid while the operator tries to move it into the central square. The speed of the circle drift is defined by different speed levels assigned in the parameter file.

#### 1.1.4 Resource management

The resource management task is a strategic fuel management task. Throughout the duration of the task, the operator must maintain the two large tanks at a set level of 2,000 fuel units while the same tanks drain at a pre-defined rate (which is adjustable in the parameter generation file). To do this, the operator uses mouse input to move fuel from one tank to another by activating or deactivating pumps. The resource management task has three primary components that need to be understood, tanks, pumps, and failure events.

A total of six tanks are available for use within the resource management task. The two large vertical tanks are the primary tanks that the operator tracks and maintains fuel levels within. The two semi-circular tanks with green backgrounds contain an infinite amount of fuel (with the drawback that pumps from this tank typically move fuel at a slower rate). The remaining two tanks start with only 1,000 units of fuel (with the benefit that pumps from this tank typically move fuel at a faster rate). The level of the smaller tanks is of no importance to the operator beyond maintaining their usage to fuel the two large tanks (i.e., if the smaller tanks run out of fuel, they will not be usable).

A total of eight pumps are available for use within the resource management task. The pumps are labeled 1 to 8, corresponding to the flow rates depicted in the center of the resource management panel. A pump's flow rate is depicted on this panel as the number of fuel units transferred per minute. Pressing one of the pump buttons will turn that pump on. Whether the pump is on is indicated by the button remaining depressed and changing color to match the fuel. Operators must strategize to use the pump flow rates correctly to maintain the fuel levels in the large tanks.

To force operators to adjust their strategies, pump shutoffs and failures occur throughout the simulation at predefined rates specified in the parameter generation file. Pump failures disable the affected pump for a specified period of time. These events severely affect the decisions and strategy employed by the operator. Pump shutoffs randomly shut off an active pump. Pump shutoffs do not disable the pump from being used, but require an active adjustment in response strategy from the operator. These events force an operator to continually adjust their mental model of the state of the resource management system and make appropriate adjustments.

#### 1.1.5 Real-time subjective workload

A measure of cognitive workload has classically been implemented in the MATB in the form of the simplified NASA-TLX (Hart and Staveland, 1988). This required the simulation to pause so that the operator could answer the 6-item scale. This would be impractical for an operational environment in which one could not be expected to answer a questionnaire with multiple items as the environment could not be paused. As an admittedly imperfect solution, a real-time subjective assessment of workload was deemed a necessary inclusion within the USAARL MATB. At a time interval set by the experimenter in the parameter generation file (e.g., every 30 s, 1 min, etc.), the subjective workload panel prompts the operator to provide a subjective appraisal of their current cognitive workload using a modified Instantaneous Self-Assessment of Workload scale (i.e., an incremental scale from 1 to 10). The operator is prompted both with a 1,000 Hertz (Hz) tone from the right speaker and a change in the color of the light next to the subjective workload slider. When this occurs, the operator has 10 s to select their workload rating on the scale. The scale then deactivates and resets itself to the 0 value until the next prompt at the predetetermined interval.

### 1.2 MATB development history

**Figure 2:**
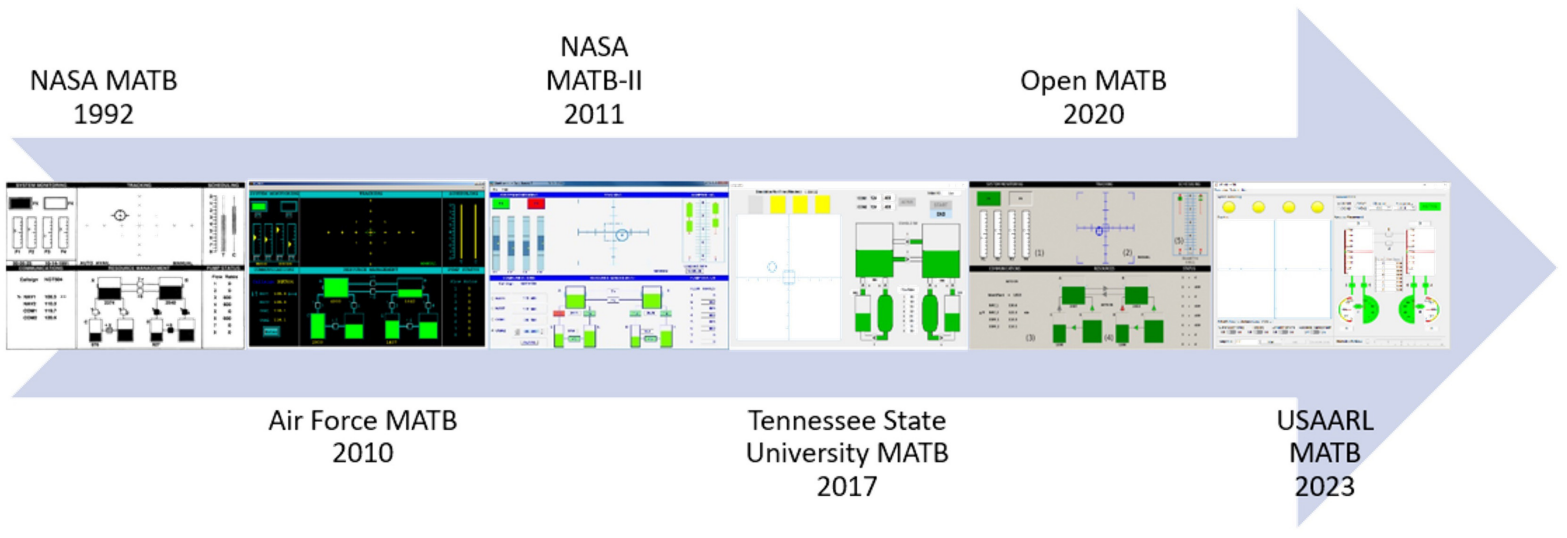
The MATB development timeline.

Since its creation, the MATB has undergone numerous iterations and development phases by a number of institutions. Figure 2 summarizes the major revisions the MATB has experienced since its creation. The MATB was originally created by Comstock and Arnegard (1992) at the National Aeronautics and Space Administration (NASA) as an assessment tool for operator workload and strategic behavior research. The MATB featured a set of four primary subtasks, displayed in black and white, that were analogous to activities performed by aviators during flight. The four tasks included a monitoring task, a tracking task, a communications task, and a resource management task. Additionally, a task scheduling display (to track the state of the tracking and communications tasks) and a subjective workload scale (NASA Task Load Index) were incorporated into the software. The program allows for operators to change the state of automation for the tracking and resource management tasks; however, automation was not implemented for the communications or system monitoring tasks. In the MATB, researchers can control the events that are shown using script files. To make these, researchers must manually specify the desired parameters and placement of each event. The MATB provided a separate output file for each task, but researchers would need to score operator performance manually as these files only provided a log of events. As the first iteration of the software, Comstock and Arnegard's (1992) MATB program laid the foundation for future iterations of the multitask battery.

Hancock et al. (1992) pioneered the initial adaptation of the NASA MATB platform. The Minnesota Universal Task Evaluation System (MINUTES) removed the communication task from the battery. This left the primary visual tasks of the MATB to be completed by subjects. These same three tasks became the basis for research platforms. Parasuraman et al. (1993) utilized the same three subtasks within the MATB but introduced modifications to allow for the automation of each component of the subtasks. Another branching research program was the Strategic Task Adaptation: Ramifications For Interface Relocation Experimentation (STARFIRE) (Hancock and Scallen, 1997). STARFIRE allowed the subtasks to be placed into a high-fidelity testing environment by overlaying the subtasks onto a three-dimensional textured environment in an effort to promote generalizability of the findings.

In 2010, the U.S. Air Force (AF) developed their own version of the MATB program; the AF-MATB (Miller, 2010). The AF-MATB aimed to capitalize on nearly 20 years of computer hardware development to update the original NASA MATB software to be compatible with modern systems. The preservation of the original appearance and features of the NASA MATB was of core importance in the development of the AF-MATB. With that being said, additional features were added to aid researchers. The AF-MATB parameter generation process enabled researchers to provide parameter specifications through a point-and-click GUI and would automatically generate event times. This dramatically reduced the time to design a simulation and posited a marked improvement over earlier iterations in that regard. Data analysis was additionally improved by the addition of a performance summary file that provided generalized performance data from operator sessions. The AF-MATB would later be updated by Miller et al. (2014) to include new functionality, including external data synchronization methods and new task modes (e.g., automated modes for all tasks, tasks could be hidden, etc.).

A year after the debut of the AF-MATB, NASA published the MATB-II program to serve as the modern update of the original NASA MATB for modern systems (Santiago-Espada et al., 2011). Again, the core subtasks were left the same to allow for efficient experiment replication. The MATB-II provided updated configuration options by implementing a graphical user interface (GUI) to control the desired modes of operation. Researchers could utilize a configuration file to modify default task parameters or select training/testing modes. To specify the sequence of events that occur during a simulation, researchers must still manually detail those in a script file. Unlike the NASA MATB which provided automated aids for the tracking and resource management tasks, the MATB-II enabled the automation of the tracking task only. The MATB-II output contains a comprehensive list of timestamps, operator inputs, and simulation events; however, grading operator performance based on the MATB-II output is somewhat inconvenient due to the lack of a performance summary file. The MATB-II was also released with eight Waveform Audio File Format (WAV) auditory messages that composed its communications task.

In 2017, researchers from Tennessee State University (TSU) identified improvements to the manner in which the MATB program operated (Thanoon et al., 2017). Under the direction of Charles D. McCurry, his students developed the core simulation loop that would comprise the four MATB subtasks using the Graphical user Interface Development Environment (GUIDE), a MATLAB-based platform. Like the original work performed by Hancock et al. (1992), the TSU-MATB marked the first modern iteration of the MATB that displayed the subtasks in a different manner while keeping the same cognitive loadings of the tasks.

The TSU-MATB was developed to enhance the MATB functionality, to make publicly available the source code, and to create the program in a software platform that is widely used in industry and academia. The availability of the source code allows researchers to make visual or audio augmentations to the subtasks using the graphical user interface. These augmentations can provide visual and audio stimuli for analysis of operator performance, workload, and human-machine interaction during the simulation (Thanoon et al., 2017). In addition, difficulty levels can be researcher-defined and analyzed for individual subtasks, and not limited to a set of pre-defined difficulty levels (Thanoon et al., 2017). Finally, the most important functionality of the TSU-MATB is access to the subtask performance in real-time during a simulation. Subtask performance metrics can be used to drive difficulty level and/or visual and audio augmentations as well as to allow for the analysis of operator performance, workload, and human-machine interaction in real-time.

Cegarra et al. (2020) then published the independently-developed OpenMATB which was programmed in Python (2.7) and included free software licensing, which allows researchers to run, use, and share modifications to the OpenMATB platform. This allows software extensibility; wherein other researchers can develop extensions and new functionality within the OpenMATB. The OpenMATB includes various feedback options, including a performance plugin in which operators are shown a 0–100 score shown visually on a bar (color depends on performance) on each task in real time. The OpenMATB, like the NASA MATB and MATB-II (Comstock and Arnegard, 1992; Santiago-Espada et al., 2011), utilizes a manual script generation process in which researchers must manually specify the events. The OpenMATB allows all four subtasks to be automated; however, there is no way to manipulate the reliability of the automatic solver which limits how automation can be utilized with the base platform. The OpenMATB additionally facilitates external data synchronization methods for studies that employ physiological measures.

### 1.3 The USAARL MATB: a novel research platform

This paper presents the U.S. Army Aeromedical Research Laboratory (USAARL) MATB. The functionality of the USAARL MATB follows that of the TSU-MATB. The USAARL MATB leverages the rich history of the MATB and the capabilities introduced by other iterations. The USAARL MATB's features were tailored around the specific research needs within USAARL but have since been developed to support general-purpose experimental designs. The introduction of the USAARL MATB here describes the functionality of the software. A user manual highlighting the functionality of each component of the USAARL MATB, is available upon request.

The purpose of the USAARL MATB is to offer the same suite of four subtasks characteristic of the classic MATB while offering general improvements for convenience and functionality. The USAARL MATB leverages the MATLAB App Developer platform to present researchers with easy-to-use GUIs for experiment design, experiment administration, and data visualization. See Figure 1 for the default USAARL MATB GUI. Development of the USAARL MATB was performed alongside the use of the NASA MATB-II and OpenMATB to identify specific areas of improvement that were necessary for an updated MATB release. The USAARL MATB includes automation that is adaptable/adaptive in nature, unique customization options (subtask variations, dynamic demand transitions), an automated parameter generation process, and improved data output and data synchronization options. These changes make the USAARL MATB a unique research platform for human performance and cognitive workload research.

## 2 Method

The USAARL MATB was developed as a progression of the TSU-MATB which was based off the AF-MATB program (Miller et al., 2014). Some changes have been made, however, to enable automation handoff functionality, improve the parameter generation process, facilitate data capture, and to improve the final data analysis methodology. This section includes a description of the system and operating requirements and an explanation of how the aforementioned goals of the USAARL MATB are achieved. This section ends by comparing the major MATB versions.

### 2.1 System and operation requirements

The USAARL MATB is a standalone desktop application that can be run on Windows, Linux, or Mac operating systems. While the USAARL MATB was developed using the MATLAB App Designer environment, it does not require an active MATLAB license to run the software. The USAARL MATB utilizes the 2018b MATLAB Runtime Library (9.5) to serve as a compiled and installable executable file. As such, no coding knowledge is needed to operate the USAARL MATB program.

One additional peripheral, a joystick, is required to interact with the USAARL MATB software (beyond standard mouse, keyboard, and display). A joystick with four individual buttons is required to interface with two of the subtasks presented in the simulation. No specific joystick model is required, as long as it has at least four buttons. A joystick calibration program is included with the USAARL MATB to map the movement and button presses of any joystick.

### 2.2 Virtual offloading guidance logic—A multi-task automation system

Each previous iteration of the MATB included task automation in some form. For instance, the NASA MATB had the option to fully automate the tracking task and resource management task only (Comstock and Arnegard, 1992). The AF-MATB, TSU-MATB, and OpenMATB have the capacity to automate every task (Cegarra et al., 2020; Miller et al., 2014; Thanoon et al., 2017). With that being said, these versions' automatic modes lack certain functionalities present in the USAARL MATB. For instance, the OpenMATB's base version does not provide a method for including imperfect automated systems (Cegarra et al., 2020). The USAARL MATB includes the VOGL system which can take control of one or more of the MATB tasks. The automation controls consist of an on/off switch for each subtask. Using their mouse, an operator can manually turn automation on or off for a specific subtask or multiple subtasks at a time. When this occurs, the VOGL system will take control of the subtask(s) and perform it to the reliability level set in the parameter generation file. Throughout the simulation, the VOGL system continuously tracks the performance of each individual subtask. If enabled in the parameter file, the automation system can provide a cue to the operator that performance has fallen below a predefined threshold and suggest the operator turn on a specific automation to assist their performance. These cues are presented as flashing lights behind the specific automation switch for which activation is suggested.

Forced automation handovers based on performance are a novel addition to the MATB that make the USAARL MATB a unique task battery for the purpose of studying adaptable/adaptive automation. Forced automation handovers can be coded into the simulation within the parameter generation file. Forced automation is defined as an automation activation that occurs without the operator's input and disables the ability of the operator to revoke control until the automation system allows. A forced automation handover occurs either at specified times or when performance drops below a specified threshold. This process turns on automation for a subtask(s) and disables the operator's ability to turn it off until reenabled by a time threshold defined in the parameter generation file or until performance has stabilized, respectively.

Each of the four subtasks have individually adjustable automation programs within the VOGL system. The reliability of the automation for each subtask can be set within the parameter file for each activation of the automation system throughout the simulation. The reliability value is a score between 0 and 100 that targets that specific value as a consistent score for the system to achieve when automation is enabled. For example, if the reliability value is set to 75 for the system monitoring task and 50 for the tracking task, the VOGL system will adjust the values of inputs to match the target response time and deviation distance to yield the respective scores for each task. The operator can visualize the performance of the automation system based on the rate and accuracy of its inputs as observed in real-time. Using this approach, different levels of automation reliability can be implemented across subtasks and adjusted over time throughout the simulation.

For the system monitoring task, the VOGL automation system can respond to the system monitoring light events that occur throughout the simulation. Currently, the VOGL automation system operates along the reaction time scoring dimension of the system monitoring task. As such, the automation will respond to light events that occur with 100% accuracy but will delay the response until a target time threshold is crossed to yield a reaction time score equal to the target reliability level. The target reaction time is derived by Formula 1:


$$\begin{array}{l} Target\;Value=Target\;Limit\;-\;\frac{Target\;Automation\;Reliability\;\times Target\;Limit}{100} \end{array}$$

Formula 1. VOGL automation system target value calculation for reliability.

The automation runs similarly for the communications task within the USAARL MATB. Again, the VOGL system scores the communications task along the reaction time metric, while accuracy is guaranteed to be 100%. The target reaction time calculation follows Formula 1, and all three aspects of the communication task are entered at the same time within the simulation loop.

For the tracking task, the VOGL automation system takes control of the input location from the joystick to minimize the distance between the input location and the target location. Turning on the automation causes the location of the controllable circle to attempt to move to the target location. However, the reliability level set by the automation causes the input location to remain at a consistent distance around the target. This provides the appearance that the system is trying to perform the task at varying levels of success.

Lastly, the resource management task also runs an automation scheme to keep operators within a specific range of values throughout the duration of being enabled. The resource management task has two primary outputs that are tracked by the VOGL automation system, tank 1 score and tank 2 score. Performance for each tank is dictated by an acceptable range of values around the target level of 2000 fuel units. To enable automation, the VOGL system allows for tank values to deviate until either tank score has surpassed a value derived by the reliability of the resource management automation (see Formula 1). Once beyond this threshold, the system will use a series of thresholds to use all available pumps to return the two tanks to the desired range. While this system seeks to derive a consistent score relative to the set reliability level, the scores are only approximate as the temporal nature and upper and lower bounds of the task restrict realistic control while also maintaining perfectly ideal scores. The VOGL system currently runs off simple target score thresholds; however, it creates a compelling experience that the system is operating at specific reliability levels.

### 2.3 Simulation loop/data synchronization

**Figure 3:**
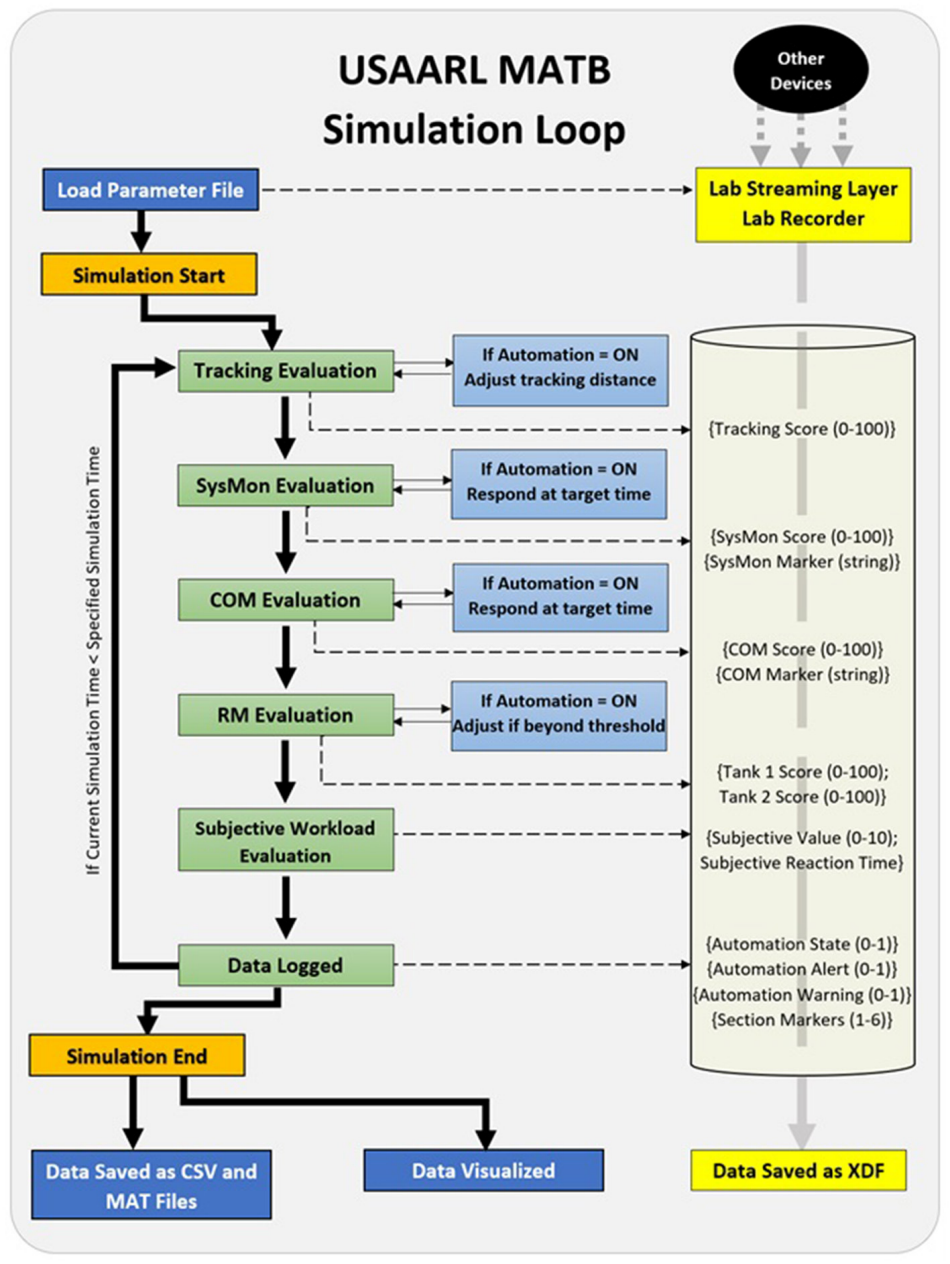
USAARL MATB simulation loop. SysMon, System Monitoring; COM, Communications; RM, Resource Management.

The USAARL MATB runs a single simulation loop while presenting the described tasks. The simulation loop runs efficiently at a typical rate of around 20 Hz on a moderately powered Windows PC platform. Each output of the USAARL MATB lists the average simulation loop time to identify the sample rate at which performance data was recorded, regardless of the computer used to run it. The simulation loop steps through each subtask, providing both updates based on the input from the user and evaluating the current state of each subtask relative to scoring thresholds set in the parameter file. Throughout this process, different options can be enabled that can change what occurs during the simulation loop. Figure 3 visualizes the simulation loop and the adjustable options available throughout the task.

Automation systems can be activated by the experimenter, as specified in the parameter file, and by the operator. Activation of the automation for any of the subtasks alters the evaluation of performance on that subtask. Further, if automation is activated, target performance set to activate changes in the subtask is also altered. How the subtask is altered can be defined in the parameter generation file. If Lab Streaming Layer (LSL) outlets are enabled within the USAARL MATB parameter file, data will be pushed to the LSL pipeline to synchronize subtasks scores, event markers, and automation states with other data (e.g., physiological data) streamed to LSL. The open-source Lab Recorder program will both visualize the USAARL MATB data streams (alongside other data streams from other software and hardware) and enable recording and saving of the resulting synchronized data. With this approach, start and stop markers from the MATB can be used to trim the peripheral data streams to only when the simulation was running, while other markers (e.g., section transitions, automation state, discrete events, etc.) can be quickly fused with other data streams.

### 2.4 Experiment parameter generation

In order to run a simulation in the MATB, certain parameters must be provided in order to set variables that control how the simulation is presented and ran, including the placement of events. Previous versions of the MATB, with the exception of the AF-MATB and the TSU-MATB, required researchers to manually create script files in order to run simulations sessions. The process of creating these script files is laborious, particularly if researchers intended to use a different simulation for each participant. The AF-MATB included a point and click GUI to make the process of designing simulations more convenient and efficient. It also allowed participants to automatically place a specified number of events for each task, such that no overlaps occurred.

**Figure 4:**
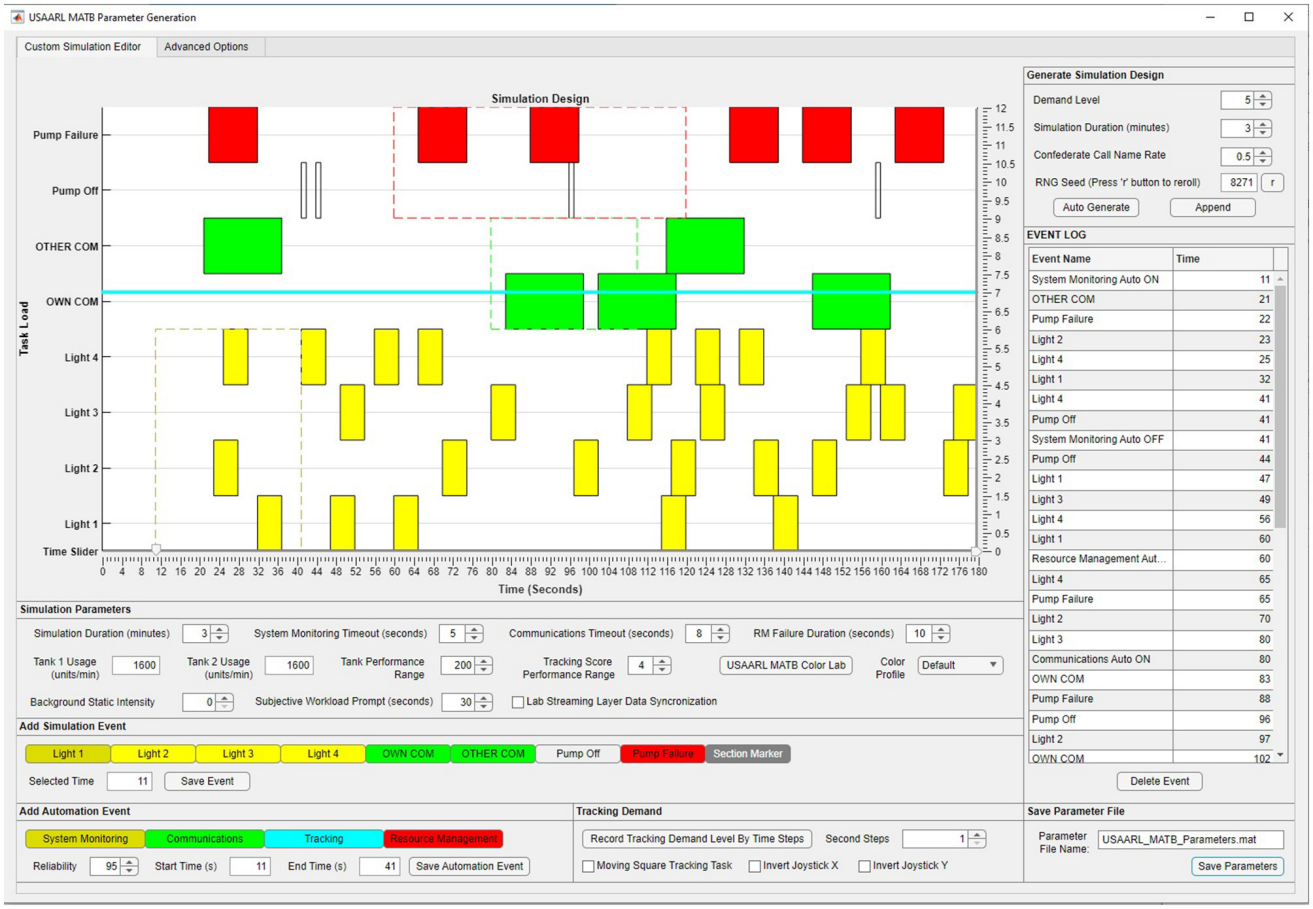
USAARL MATB parameter generation GUI.

The USAARL MATB implements a parameter generation system similar to the AF-MATB with several noteworthy modifications. One change is that the placement of events can be seen visually within the Parameter Generation GUI (see Figure 4). This change enables researchers to quickly assess whether events are well-distributed over the course of the session as well as to easily display the placement of events so that other researchers can replicate the study. Experimental replicability is further facilitated by the inclusion of seed generation. Instead of having to provide the full script file, researchers can enable other researchers to replicate their exact event placement by providing the parameter settings and seed.

The USAARL MATB provides a plethora of customization options within the Parameter Generation GUI, with some that were not included in the AF-MATB or TSU-MATB (Miller et al., 2014; Thanoon et al., 2017). These customization options facilitate an array of experiments ranging from cognitive workload assessments, visual attention modeling, performance modeling, and automation dynamics, among others. These options are discussed further in the following section.

### 2.5 Customization options

Along with allowing convenient generation of parameters, another goal of the USAARL MATB is to enable researchers to customize the original MATB to allow for new experimental designs. Every previous version of the MATB includes some basic options for customizing the functioning of the four main subtasks and the USAARL MATB is no exception (Cegarra et al., 2020; Comstock and Arnegard, 1992; Miller et al., 2014; Santiago-Espada et al., 2011; Thanoon et al., 2017). For instance, researchers can set a simulation duration and demand level that fits the needs of their simulation. The USAARL MATB also allows the options to provide live performance feedback to operators, and to customize the color palette of the simulation GUI.

#### 2.5.1 Basic customization options

Every previous version of the MATB includes some basic options for customizing the functionality of the four main subtasks and the USAARL MATB does as well (Cegarra et al., 2020; Comstock and Arnegard, 1992; Miller et al., 2014; Santiago-Espada et al., 2011; Thanoon et al., 2017). The USAARL MATB includes basic customization options that influence how the specific tasks function.

Some general options that researchers can set for a simulation include specifying the simulation duration and demand level (controls how many events will be shown for each task). Demand transitions can be included by “appending” a new set of generated events onto an existing set of events. Researchers can also specify how often the subjective workload assessment will be shown. The USAARL MATB includes automation options that were not available in previous iterations of the MATB. Within the Parameter Generation GUI, researchers can control what the reliability level of the VOGL system is for each task, set times where the automated system will take control of each task, and specify whether the operator can initiate a transfer of control over to the automation.

For the system monitoring task, researchers can set the timeout value, indicating how long operators have to respond to a system monitoring event. The communications task also has a timeout value, while also allowing researchers to change the confederate call name rate, increasing the amount of distractor call names, or enabling background chatter COM in which the distractor call names are replaced by a static noise of a volume specified by the researcher. For the tracking task, researchers can specify a distance from the center that constitutes a performance value of zero (performance range). In addition, researchers can adjust the speed that the circle drifts, invert the X and/or Y axes of the joystick, and change the task orientation by allowing operator control of the target square to move to the drifting circle's location. For the resource management task, researchers can alter the flow rates of the various pumps, change the length of a pump failure (when a pump cannot be used), and set the performance range for the task.

#### 2.5.2 Feedback information

Like some of the more modern versions of the MATB such as the TSU-MATB or OpenMATB platforms (Cegarra et al., 2020; Thanoon et al., 2017), the USAARL MATB includes options by which operators can be provided with live performance feedback. With that being said, the USAARL MATB's primary feedback option is somewhat different from those in the TSU-MATB or OpenMATB in that they are intended to be used, at least in some cases, as a cue to enable automation.

When task cueing is enabled for a given task, researchers set a threshold level of performance for that task. If an operator's performance for a task falls below that threshold value, they will be prompted to enable automation for that task. The background of the automation panel associated with the relevant task will blink red until the average performance for that task increases beyond the threshold.

#### 2.5.3 USAARL MATB color lab

The NASA MATB was in black and white (Comstock and Arnegard, 1992). While other versions of the MATB since then were colorized, they did not include the option to customize color palettes (Cegarra et al., 2020; Miller et al., 2014; Santiago-Espada et al., 2011; Thanoon et al., 2017).

**Figure 5:**
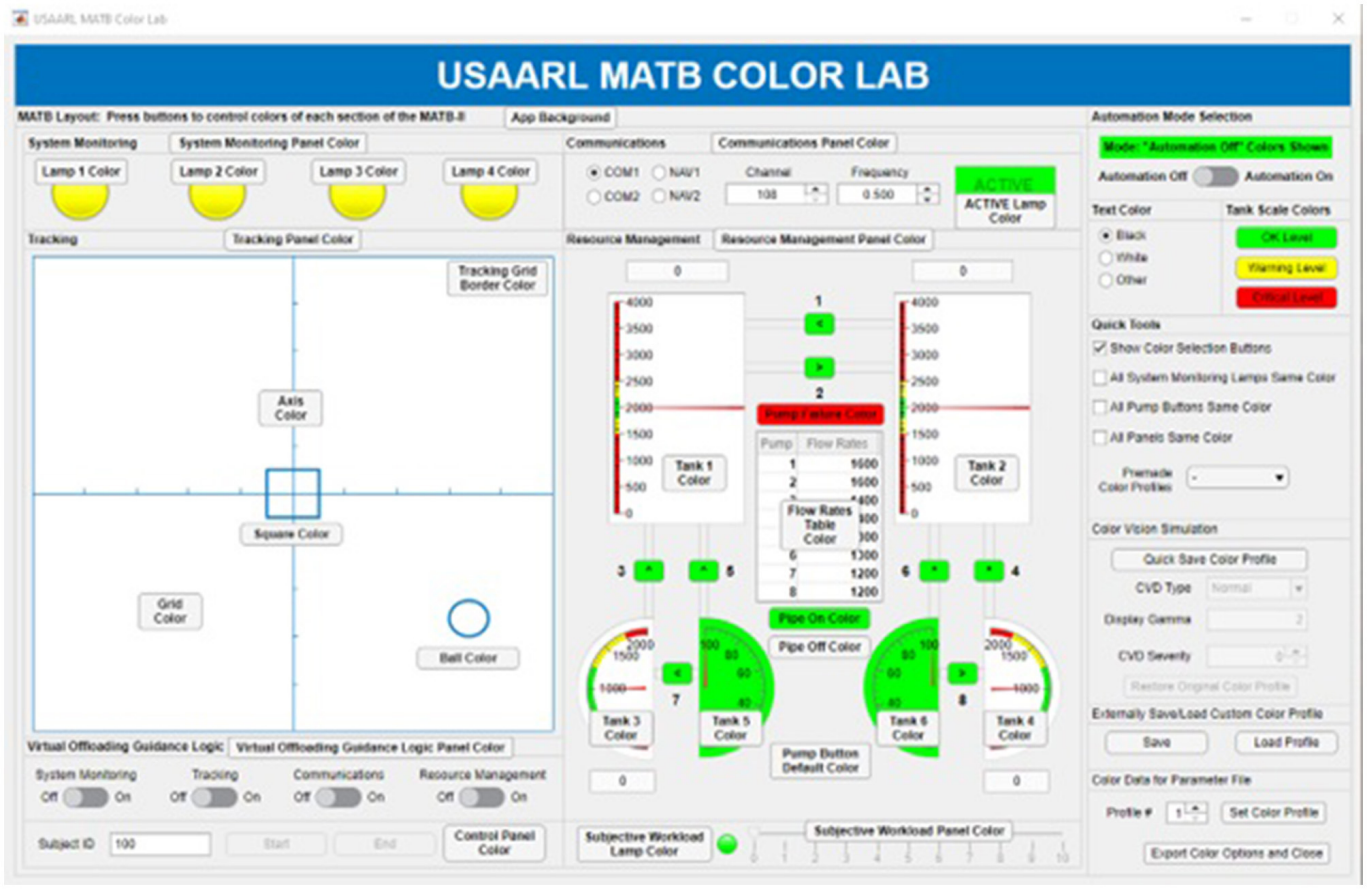
USAARL MATB color lab GUI.

The USAARL MATB offers an additional parameter generation program (that can be accessed through the “Simulation Parameters” panel of the Parameter Generation GUI) to control how color information is presented to a subject. This program is called the MATB Color Lab and is depicted in Figure 5.

Nearly all aspects of color within the USAARL MATB can be controlled by the MATB Color Lab application. A point-and-click style GUI was designed to allow researchers to control the color of each element of the MATB. Clicking a button next to a specific element of the MATB allows researchers to input a new color using a GUI-based color menu. The sidebar on the right contains more options, such as automation color controls, quick color profiles, color vision deficiency simulation, and allows saving color profiles for future use. The USAARL MATB color vision model, modified from a model developed by Machado et al. (2009) and expanded by Harding et al. (2020), allows researchers to simulate color vision deficiency. The USAARL MATB Color Lab can be used to examine how color changes in the GUI impact performance, to simulate color vision deficiency, or to create simulations that accommodate those with color vision deficiency.

### 2.6 USAARL MATB data output

The USAARL MATB provides data in easy-to-use Excel and MATLAB formats. Many previous iterations of the MATB provided data logs with a record of every event that took place during the simulation along with timestamps (Comstock and Arnegard, 1992; Santiago-Espada et al., 2011; Miller et al., 2014; Thanoon et al., 2017; Cegarra et al., 2020). These data output files, while rich with information, could be very inconvenient for researchers to process. The AF-MATB provides a partial solution to this problem by including a performance summary file in which higher-order information from simulation sessions; however, researchers that want to examine changes in performance or workload over time would still have to parse through the event log (Miller et al., 2014). Further improvements to the data output and data analysis process were a necessary inclusion for an updated MATB release.

The USAARL MATB includes an improved internal data output system over these previous iterations in that comprehensive information from trials (system state, performance scores, etc.) is provided in an immediately processable form. This is possible because the system state and performance are assessed in every iteration of the USAARL MATB Simulation Loop. Raw data (i.e., every sample collected during each simulation loop), average data, and scaled performance scores, i.e., normalized between 0 and 100 relative to experimenter defined thresholds, for each subtask are available in the output. All the raw data is saved in the corresponding CSV file. A summary of the data dictionary defining the USAARL MATB data outputs is provided in Table 1.

**Table 1:** USAARL MATB data output descriptions.

**Data output name**	**Data type**	**Description**
System monitoring RT	Seconds	Reaction time of joystick button press.
System monitoring score	Normalized score	$\frac{SystemMonitoringTimeout-SystemMonitoringRT}{SystemMonitoringTimeout}$ × 100
System monitoring accuracy	Percentage	Accuracy of system monitoring button presses. Pressing incorrect buttons yield errors.
Communications RT	Seconds	Reaction time of a correct response to a communications prompt.
Communications score	Normalized score	$\frac{CommunicationsTimeout-CommunicationsRT}{CommunicationsTimeout}$ × 100
Communications accuracy	Percentage	Number of elements correct (out of three elements) for a communications response.
Tracking deviation	Graph units	Distance between the center of the target and user-controlled element.
Tracking score	Normalized score	$\frac{TrackingDeviation}{TrackingRange}\times 100$
Resource management tank 1 value	Integer	Raw tank value of Tank 1.
Resource management tank 1 score	Normalized score	$\frac{RMTank1Value-2000}{RMRange}\times 100$
Resource management tank 2 value	Integer	Raw tank value of Tank 2.
Resource management tank 2 score	Normalized score	$\frac{RMTank2Value-2000}{RMRange}\times 100$
Subjective workload value	Integer	Subjective workload value input by the operator.
Subjective workload RT	Seconds	Reaction time to the subjective workload prompt.
Automation state	Matrix	A logic matrix indicating whether an automation system for a subtask was active (1) or inactive (0).
Discrete task loading	Matrix	A matrix indicating how many discrete tasks are loading on the operator.
Event markers	String	Descriptions of events occurring in the USAARL MATB program (e.g., “Start,” “Stop,” “COM,” etc.).

### 2.7 Direct comparison

Throughout this section, we compared the capabilities of the USAARL MATB with some of the MATB's previous iterations. A direct comparison of some of the features available in the different versions of the MATB is provided in Table 2.

**Table 2:** Direct comparison of MATB features.

	**Capability**	**MATB (Comstock and Arnegard, 1992)**	**MATB-II (Santiago-Espada et al., 2011)**	**AF-MATB (Miller et al., 2014)**	**TSU-MATB (Thanoon et al., 2017)**	**OpenMATB (Cegarra et al., 2020)**	**USAARL MATB**
Parameter generation	Automatically generate event placement.	×	×	3	3	×	3
	Visualize events in GUI.	×	×	×	3	×	3
	Parameters replicable by seed.	×	×	×	×	×	3
Automation	Some amount of task automation included.	3	3	3	3	3	3
	All tasks have automated modes.	×	×	3	3	3	3
	Automation reliability can be directly manipulated.	×	×	3	3	×	3
	Forced automation handoffs based on operator performance.	×	×	×	×	×	3
Customization	Basic task customization (i.e., timeouts, pump values, etc.).	3	3	3	3	3	3
	Ability to provide feedback information to operator.	×	×	×	3	3	3
	Ability to change the color of all GUI elements.	×	×	×	×	×	3
Data output	Provides a summary file with performance for each task.	×	×	3	×	×	3
	Minimal data cleaning required for a basic simulation.	×	×	×	×	×	3
	Synchronization with external devices.	×	×	3	3	3	3
Availability	Source code availability.	3	×	×	×	3	×

## 3 Results

The core idea behind the creation of the USAARL MATB is to enable additional functionality and data processing efficiency beyond that presented by the original MATB-II program. As such, the USAARL MATB was coded to deliver customization of multiple parameters of the task in a manner that is understandable by non-programmer researchers. The point-and-click GUI of the USAARL MATB allows for efficient sharing and building of different types of experiments examining factors from cognitive workload assessment, automation interventions, and dynamic demand transitions. This section will review some exemplar use-cases that can leverage the functionality of the USAARL MATB to enable sophisticated examinations of these research areas.

### 3.1 Composite cognitive workload assessment

Cognitive workload results from the subjectively experienced physiological phenomenon of the interaction between an operator's available cognitive resources and the demands of a task (and/or environment) (Van Acker et al., 2018). Cognitive workload assessment studies utilize three categories of measurement techniques to determine the level of cognitive workload experienced by an operator. These techniques include performance, subjective, and physiological metrics. The USAARL MATB was designed with the ability to capture all these metrics and synchronize the output with the integration of LSL.

The USAARL MATB captures the operator's performance throughout the simulation across the four tasks. The performance data is threaded with additional markers of events that occur within the task (e.g., start and stop markers, section markers, discrete events) as well as the discrete task load that occurs during each simulation loop. These markers allow for efficient synchronization with common physiological indicators of cognitive workload. Physiological data can be synchronized alongside performance and subjective data using Lab Streaming Layer.

### 3.2 Automation handoffs

The subtask automation capabilities within the USAARL MATB provide means to assess how cognitively offloading a subtask to automated systems influences performance, subjective, and physiological metrics. The USAARL MATB provides means for answering the questions of when, how, and what to automate to most efficiently aid the operator. Using performance-driven adaptive automation, researchers can modify when automation takes control of a subtask or guide when the automation system alerts an operator to turn on automation. Automation can be handed over in either a voluntary manual manner or in a forced automation takeover to identify how cognitive offloading should occur. Lastly, what should be automated can be modulated through task parameters and cognitive task analysis of each individual subtask to determine performance enhancements that occur as a function of offloading a specific task.

### 3.3 Trust in automation

Examining how operators trust in and rely on imperfect automation systems is a key area of research focus (Parasuraman and Riley, 1997). The USAARL MATB offers researchers the ability to manipulate the reliability of the automation of each subtask to create scenarios that are ideal for research regarding operator trust in automation. Automation reliability can be adjusted between and within simulations, offering a unique capability to simulate a progressively failing or improving automated system. Additionally, performance-based adaptive automation cues built into the USAARL MATB enable assistive cues as to when automation should be enabled using performance-driven adaptive automation processes. Examining how operators choose to enable automation both on their own volition and when prompted can provide insights regarding operator trust in automation.

### 3.4 Machine learning

The capture and synchronization of physiological and performance data provides researchers the ability to investigate machine learning in research utilizing the USAARL MATB. Machine learning methodologies could be used for cognitive workload predictions, strategies to engage automation handoffs, and performance predictions based on multi-task demands. These classifications and predictions can be used to aid in multitasking training, automation development, and experimental design.

### 3.5 Performance modeling

The performance metrics captured by the USAARL MATB allow for sophisticated modeling of task demand and individual differences. A study on performance modeling through gender-based evaluation utilizing the USAARL MATB was presented at the 2024 Industry, Engineering, and Management Systems (IEMS) conference. This study emphasized the importance of understanding how individual differences and perceptions of workload impact task performance (Adeyemi and Bommer, 2024).

The USAARL MATB Color Lab allows for precision control of GUI color changes throughout an experiment to examine how the color of displayed information can affect performance. A pilot study, presented at the Industrial Engineering and Operations Management (IEOM) conference in 2023, examined the impact of color in GUIs on human performance using the USAARL MATB. The primary aim was to validate the proposed experimental design and methodology and pinpoint any necessary adjustments for achieving reliable results. Statistical differences between different scenarios presented with a variation of background colors and task complexities were observed using the tools available in the USAARL MATB (Adeyemi and Bommer, 2023).

## 4 Discussion

The USAARL MATB is poised to serve as a valuable research tool for researchers needing an experimental platform with a rich, validated history and that also enables simple experiment creation and customization, simulation of real-world applicable flight tasks, and efficient to process and analyze data output. This platform is created to serve as a standalone application that can be easily transferred between researchers to allow for replication and iteratively developed studies. Additionally, the operational relevance of the subtasks within the MATB is an important factor when considering early experimental design in a research program.

Some limitations of the USAARL MATB in its current iteration include a lack of open access, the reliance on the tools made available by the MATLAB platform, and a lack of data sets currently available for testing and analysis. Currently, the USAARL MATB is a stand-alone executable file. While this approach allows for simple sharing and execution of the USAARL MATB between researchers without a programming background, it does limit future development outside of the USAARL organization to those without access to a MATLAB license. While the MATLAB platform offers a large array of toolboxes to meet the needs of the USAARL MATB, there are some aspects of individual component control that are limited by the platform. Circumventing these limits may require updates to new releases of MATLAB periodically in order to meet the needs of researchers using the USAARL MATB. Lastly, as the USAARL MATB is relatively new, there are only a few datasets available to use for future developments. As more studies are conducted and finalized, the collected data will be used to further validate the USAARL MATB components.

As the USAARL MATB is further developed, these limitations and other additions to improve usability and functionality will be implemented. A wide assortment of studies at USAARL are currently incorporating the USAARL MATB as a laboratory testbed, which will undoubtably lead to continued refinement of the software. Future updates are planned based on the domains of research presented in this article. Machine learning models are currently being developed using the USAARL MATB to predict the cognitive state of the operator based on performance and physiological data. More advanced automation reliability and handoff schemes are being explored to further enhance the efficiency and validity of the VOGL automation system. Lastly, additional task modifications and mission sets are being tested for inclusion with the USAARL MATB program in future releases. Current efforts have focused on validating a diagnostic multitasking metric assessed in the USAARL MATB, adding in digital competitors and support agents utilizing Chat Generative Pre-Trained Transformer (ChatGPT) elements, and interlacing information from one task to aid in the performance of another task.

The current release of the USAARL MATB, detailed in this publication, is version 2.2. Additionally, interested researchers may contact the authors to obtain the software and assistance in its operation and data analysis. The USAARL MATB software is accompanied by a user manual that details each individual component of the software in more depth.

## Data Availability

The original contributions presented in the study are included in the article/supplementary material, further inquiries can be directed to the corresponding author.
